# The specifics of applying systemic psychotherapy to team sports games

**DOI:** 10.3389/fpsyg.2025.1534306

**Published:** 2025-03-19

**Authors:** Paweł Adam Piepiora, Ligiana Mihaela Petre, Jolita Vveinhardt

**Affiliations:** ^1^Faculty of Physical Education and Sports, Wroclaw University of Health and Sport Sciences, Wrocław, Poland; ^2^Faculty of Psychology and Educational Sciences, University of Bucharest, Bucharest, Romania; ^3^Institute of Sport Science and Innovations, Lithuanian Sports University, Kaunas, Lithuania

**Keywords:** personality in sports, team dynamics, team sports games, top athletes, subclinical narcissism, systemic psychotherapy

## Abstract

High-level team sports competition creates considerable degree of mental workload for the players, particularly in teams with prominent players displaying characteristics of subclinical narcissism. This affects team effectiveness, which is contingent upon the harmonization of the players’ personalities. This perspective examines the specific application of systemic psychotherapy in team sports games. We analyze key factors including team compositions, therapeutic processes, and the factors that contribute to the onset and persistence of pathological symptoms. Our analysis reveals that while systemic psychotherapy effectively addresses team dynamics by treating the team as a unified system, its success depends on multiple variables that influence treatment outcomes. Understanding these specifics enables more effective implementation of systemic psychotherapy in a team sports, though its effectiveness is not constrained by universal patterns. This perspective contributes to expanding the therapeutic approaches in competitive team sports.

## Introduction

Contemporary team sports games represent a complex social phenomenon (Buenemann et al., 2023), where top athletes function as pop-culture icons (Coffee et al., 2015), influencing behavioral norms especially among young fans (Simsek and Ozturk, 2024). Top athletes, like ancient heroes, are popular and adored, which is certainly part of those experiences that shape their personality (Spielmann et al., 2022). But in cases of sporting failure, the effects of fans’ attributional egoism are noticeable among them. From one day to the next, top athletes become objects of heckling and hate speech, with not all of them mentally resilient enough to cope (Kago and Venkataraman, 2023). Added to these psychological burdens on athletes is the constant threat of exclusion. A particular player’s poor level of play can be a reason for their exclusion from a leading team, which relates strictly to their functioning under pressure for fear of keeping their contract or securing a new one (Jekauc et al., 2024).

Within teams, top players often display subclinical narcissism (Spielmann et al., 2024) creating what literature terms the ‘star player dilemma’ – where heightened self-esteem and performance abilities create both advantages and team dynamics challenges (Apitzsch, 2019). It should be noted that there are also teams with several such ‘stars’ in the first line-up and teams with only ‘stars.’ In such cases, these teams should, according to the statistics, function very well in and out of the game. But one notices an inverse relationship that translates into average or poor functioning of these teams in and out of the game (Filho, 2021). Leaving aside the on-field skills of the players, positive cooperation will depend on the chemistry of the players’ personalities (Wergin et al., 2018). The problem here is a socially prevalent phenomenon in simple terms - I like you, so I trust you - which translates into field situations for a given team. Meanwhile, in team sports games, the desirable phenomenon is in terms of - I may not like you, but I trust you–which translates into the effectiveness of the whole team’s conduct of the game according to the principle that we all play to one goal (Wergin et al., 2019).

The method for developing this team effectiveness is psychotherapy, which in a sporting environment is a form of guidance, education, and interpersonal persuasion. Here, the psychotherapist accompanies the team during the change, helps to modify the team’s attitudes and influences them (Lee et al., 2024). This is achieved through purposeful and planned psychological interactions that lead to the alleviation or removal of the symptoms of subclinical narcissism of the ‘star’ and the improvement of the psychological and social functioning of the whole team supporting their aspirations to stay healthy and grow (Chammas et al., 2024). It is also important to note that psychotherapy can also be applied for self-development purposes to athletes without psychopathological symptoms. But it should be noted that developing universal methods to effectively help an athlete in need is not a straightforward process (Wilkie et al., 2024). Since the late 1940s, research has been ongoing into how psychotherapy should be conducted and what type of change can be considered as an indicator of contemporary effective psychotherapy in sport (Abdelhalim, 2023). In the environment of team sports games - collaborating with teams - the high effectiveness of systemic psychotherapy is noted, which translates into good functioning of athletes in and out of competition (Brenner et al., 2023). This article aims to examine the specific application of systemic psychotherapy in team sports, focusing on how it address team structure, interpersonal dynamics, and the professional environment. In the article it has been proposed that systemic psychotherapy offers an adequate framework for understanding and improving team functioning beyond individual performance enhancement.

## Specifics of the application of systemic psychotherapy in sports team games

Systemic psychotherapy in team sports requires specific adaptations of traditional therapeutic approaches to address the unique dynamics of athletic teams. The following section outlines the key components and considerations in this specialized application.

Systemic psychotherapy is a form of group therapy that is successfully applied to team sports games. The facilitator does not allow mutual interpretations of the team members, as he/she works sequentially with the individual athletes in the group. The others are participating observers if they are not involved in the setting (McKeown et al., 2024). The psychotherapist recognizes attitudes of individuality, kindness, respect, and care. He/she allows the following settings of the players in therapy: in a circle; in relation to the field position - forwards, midfielders, defenders, goalkeepers; in cases of thwarted relationships of an athlete to another athlete. The coach thus makes it clear that he or she believes in the team as participants in the therapy. He or she sends a non-verbal message that the athlete can handle himself or herself, reinforcing the athlete’s self-image with this treatment (D'Ascenzo et al., 2024). The therapeutic setting is structured to reflect both team hierarchy and field positions, with specific arrangements (circular, positional, or relationship-based) serving different therapeutic purposes. While working sequentially with individual athletes, the therapist maintains group engagement by positioning others as active observers. This approach combines individual focus with team awareness, essential for addressing both personal and collective dynamics (Erasmus et al., 2025).

It should be emphasized that non-verbal communication in this therapeutic relationship serves to: externalize and express feelings and emotions; regulate the interaction; reinforce and confirm the verbal message; maintain the self-image; maintain contact (Eram et al., 2022). Among the factors responsible for the emergence and persistence of pathological symptoms, systemic therapists in team sports games mainly mention: disruptions of the team structure, disruptions of communication within the team, and abnormalities related to the rules governing team functioning. Pathological aspects of the system structure include disruptions in the roles played by individual team members and boundaries between subsystems (Prusiński, 2024).

As for the organization of the therapy sessions, the captain first selects representatives–the main players of the team, from among the psychotherapy participants. Then, in a focused manner, he positions them in relation to each other, according to his intuition regarding where they should sit (Crits-Christoph et al., 2020). It is important that the chosen representatives listen carefully to how they feel about the place they are in. The norm is that reactions may vary, as is usual among the team members concerned. But they allow an observation of the hidden dynamics, where facts play a key role (Culina et al., 2023). This spatial organization serves multiple purposes: it reveals implicit team hierarchies, highlights relationship patterns, and provides a physical representation of team dynamics. The varying reactions of players to their positions often uncover underlying tensions or alliances that might not be apparent in regular team interactions.

Systemic psychotherapy in team sports games generally emphasizes understanding the abnormalities of a given team’s relationships, as well as the ways in which they train and play. Invariably, however, treatment encompasses the entire team system. By which the focus of work in systemic psychotherapy is change in the functioning of the players (Hermanowski, 2023). It is most often a short-term psychotherapy aimed at solving a current problem in the team in question. It deals with the observed, current behavior of the players and the effects of this behavior (Lisica et al., 2023). Therefore, the psychotherapist actively and directive helps the athletes to identify their current difficulties (Gelso, 2014). It has been possible to identify specific factors of systemic psychotherapy in team sports games on the basis of an analysis of the players’ life experiences and accompanying emotional experiences. This is the unwinding of repressed emotions, the release from the compulsion to indulge in these emotions, and the acceptance of one’s past emotional experiences and distinguishing them from the feelings that current events evoke (Ströhle et al., 2022).

It should be emphasized that the solutions of systemic psychotherapy in team sports games are obtained after many stages and changes. It is important that the players, seeing the solution to the problem, internalize it as much as they can. The mechanism should be as follows from getting the problem by internalizing the problem solution to the healing process (Ekelund et al., 2022). At the same time, it takes a long time to achieve full wellbeing. The systemic psychotherapist, by ‘digging’ in the mind of the athletes, always brings something to light (Stillman et al., 2016). Their task is to name the facts openly, even if these are painful for the team members. Therefore, the psychotherapist listens to what the athletes say and openly names it. In this way, he/she gives clues to the athletes that they can use and take action to change a difficult situation (Lundqvist et al., 2024). Sometimes, however, the psychotherapist gives messages to the team along the lines of ‘what else is there to explain?’ or ‘this cannot be fixed’. These types of messages should be read as definitive. They are messages that nothing can be changed or fixed in each situation. Not every player is ready to hear this kind of statement during psychotherapy (Ströhle, 2019). Here, the psychotherapist does not ask about emotions, but only relies on facts. Therefore, finding oneself in a specific setting triggers strong emotional reactions in the athletes and allows them to make insights into their own emotions, thoughts and actions. This reaction stimulates athletes to reflect further and makes them reflect (Schenk and Miltenberger, 2019).

Accordingly, therapeutic factors have been derived for systemic psychotherapy in team sports games teams. These are the accepting attitude of the psychotherapist towards the team members. It is also the non-verbally transmitted messages that the psychotherapist believes in the team. Furthermore, it is the psychotherapist’s actions by giving impulses to the team to act, giving them time to act and not interfering with the feelings and thoughts of the players (Bartholomew and Lockard, 2018). The above-mentioned healing factors are implemented by the psychotherapist by naming interaction patterns and encouraging the team to act, where he or she uses a positive connotation and reformulates the evaluation of the phenomenon in question. This is augmented by proposing specific behaviors for team members in the form of injunctions and advice. And interventions here can be paradoxical, i.e., the content of the recommendation is usually the opposite of the expected response of the team (Levitt et al., 2016).

## Discussion

Systemic psychotherapy interventions and approaches in team sports games aim to mitigate the negative impact of socially aversive traits in teams, while potentially harnessing any positive aspects of the system for improved psychological functioning and player well-being (Aigner et al., 2024). This is a systems psychotherapy approach taken from family therapy that has been transferred to the sports team environment (Tramonti et al., 2024). The effectiveness of this framework stems from its ability to address team system through a familial lens, recognizing patterns of interaction, hierarchies, and emotional bonds that mirror family structures. In this view, the head of the family-team is the captain, and the players subordinated to him who are important to the team system. On the other hand, it should be emphasized that the effectiveness of team system psychotherapy will depend on several variables that must be carefully considered in its implementation (DeRubeis et al., 2014).

Firstly, the composition of the team is important (Piepiora, 2021). If there is only one top player on a given team in relation to the rest of the system, the therapeutic work proceeds differently when the ‘star’ socially identified as the team leader is also the team captain, and differently when the top player and leader in one does not act as team captain. Furthermore, the situation breaks down even more differently for therapeutic work when there are several ‘stars’ on the team or the entire system is made up of such individuals. In such cases, the psychotherapist must reckon that his or her work is with athletes with strongly formed personalities, and that the captain - the ‘star’ - is not always perceived in the team as the team leader.

Secondly, the timing of systemic psychotherapy is important (Kazdin, 2009). At the beginning of the work with the team, the psychotherapist predetermines the time to work in this system. But from session to session increasingly difficult and serious problems in the functioning of the team may come out. It is often the case that the team’s systemic psychotherapy is planned as a short-term therapy but transforms into a long-term psychotherapy due to the specific problems of the players’ functioning in the system. Which means that the achievement of the effects of the therapeutic factors of these interventions can be postponed over time and that it cannot be clearly determined that the planned goal will be achieved within a rigid time limit. It should be emphasized that the above issue does not always involve the understanding of the sports associations, managers and team owners concerned. The sustained lack of on-field effectiveness of a team participating in systemic psychotherapy often translates into decisions to discontinue this psychotherapy, which is not a solution to the problem of how the system works.

Thirdly, the team’s sense of efficacy is important (Hyndman et al., 2024). In systemic psychotherapy, the therapist collaborates with teams whose daily functioning is subordinated to macro-cycles, mesocycles and micro-cycles of training. This means that, depending on the given preparation, start and transition periods, the effects of the systemic psychotherapy conducted in the respective team may be distributed differently. And on these effects will always depend on the team’s sense of efficacy, which is directly linked to the quality of the team’s play. The team’s sense of efficacy is made up of variables such as the importance of the game; the field tasks that the players have to perform; the composition of the team on the field; the composition of the opposing team on the field; the experience of the players; the training levels of the players; the specific tactics of the teams in the respective team sports games; the individual characteristics of the athletes. This means that, depending on the variables described above, any team that is in systemic psychotherapy will feel confident in the game.

Fourthly, the constant composition of the team is important (CraigJavier and Fernandez-Navarro, 2024). If a team that participates in systemic psychotherapy has a constant composition of players in each season or over several consecutive seasons, then the effectiveness of the therapeutic work is achieved more quickly, and the effects are long-term. This is particularly evident in high-class club teams and national teams of other countries in various team sports games. The personality building of teams with fixed squads is always a staggered process, but once the goals of systemic psychotherapy have been achieved, these are teams that function well over an extended period and triumph in the games.

Fifthly, collaborating with player resistance is important (Marken and Carey, 2015). In systemic team psychotherapy, resistance can stem from deep-rooted team myths - for example: ‘with this team we will definitely not win, so we will play for as little embarrassment as possible’; and abnormally formed boundaries - for example: the top players on the team respect only themselves, and display behavior of subclinical narcissism towards the rest of the players. It is important to emphasize that systemic psychotherapy aims to work through issues that hinder communication or modify the team structure so that the team functions properly. But each player will manifest resistance differently. Some of the most common examples of resistance among athletes include behaviors such as: absence from sessions (due to forgetfulness, illness, other plans), frequent lateness, silence during sessions, difficulty in expressing one’s thoughts, verbosity preventing dialogue, hiding important issues, avoiding important topics during sessions, avoiding responsibility for the session, changing the topic when discussing important issues, making digressions unrelated to the topic, frequently saying ‘I do not know’ in response to the therapist’s comments and questions, breaking rules and principles established at the beginning of therapy, maintaining ‘star’ status, avoiding to speak directly about emotions in the therapeutic relationship.

Sixthly, catharsis is important (Greenberg, 2014). These are corrective emotional experiences that have a bearing on the personality chemistry of the players in the team. They will be manifested through the players’ emotional detachment and their release from experienced feelings. As a result of the release of emotions through catharsis, a decrease in mental tension, a decrease in ruminative power, an increase in the cognitive analysis of experiences allowing them to influence feelings, the use of life experiences to solve current problems, the restoration of the ability to recognize one’s feelings and use them for one’s own actions, and a shared responsibility for the team and relationships within it are noted among athletes.

These multiple dimensions of systemic psychotherapy in sports teams - from team composition and timing to resistance and catharsis - highlight both its potential and its complexities. Successfully navigating these elements requires not only therapeutic expertise but also a deep understanding of sports team dynamics and the pressures of competitive environments.

### Practical recommendations

This perspective presents distinct applications for both psychotherapists and coaches. For psychotherapists, it demonstrates how systemic psychotherapy can be effectively adapted to team sports games, focusing on team hierarchy, dynamics, and performance cycles. For coaches, it provides a framework for understanding team psychological development and integrating systemic principles into team building. This approach indicates that beyond sport psychology and sport psychiatry, there is still the potentiality of using psychotherapy for sport.

## Conclusion

Systemic psychotherapy demonstrated validity in team sports games through its approach to team-as-system dynamics. However, the efficacy of team functioning cannot be reduced to general patterns, and the attainment of desired outcomes occurring within a short-term or long-term framework. This creates tension with competitive sport’s emphasis on immediate results, suggesting the need for balanced expectations between therapeutic process and performance demands.

## Data Availability

The original contributions presented in the study are included in the article/supplementary material, further inquiries can be directed to the corresponding author/s.
